# Brain perfusion in dementia with Lewy bodies and Alzheimer’s disease: an arterial spin labeling MRI study on prodromal and mild dementia stages

**DOI:** 10.1186/s13195-016-0196-8

**Published:** 2016-07-12

**Authors:** Daniel Roquet, Marion Sourty, Anne Botzung, Jean-Paul Armspach, Frédéric Blanc

**Affiliations:** ICube laboratory, University of Strasbourg, CNRS, FMTS(Fédération de Médecine Translationnelle de Strasbourg), ICube – IPB, Faculté de Médecine, 4 rue Kirschleger, Strasbourg, 67085 France; University Hospital of Strasbourg, CMRR (Memory Resources and Research Centre), Strasbourg, France

**Keywords:** Cerebral blood flow, Prodromal phase, MRI, Early diagnosis, Insula

## Abstract

**Background:**

We aimed to describe specific changes in brain perfusion in patients with dementia with Lewy bodies (DLB) at both the prodromal (also called mild cognitive impairment) and mild dementia stages, relative to patients with Alzheimer’s disease (AD) and controls.

**Methods:**

Altogether, 96 participants in five groups (prodromal DLB, prodromal AD, DLB with mild dementia, AD with mild dementia, and healthy elderly controls) took part in an arterial spin labeling MRI study. Three analyses were performed: a global perfusion value comparison, a voxel-wise analysis of both absolute and relative perfusion, and a linear discriminant analysis. These were used to assess the global decrease in perfusion, regional changes, and the sensitivity and specificity of these changes.

**Results:**

Patterns of perfusion in DLB differed from AD and controls in both the prodromal stage and dementia, DLB having more deficits in frontal, insular, and temporal cortices whereas AD showed reduced perfusion in parietal and parietotemporal cortices. Decreases but also increases of perfusion in DLB relative to controls were observed in both absolute and relative measurements. All these regional changes of perfusion classified DLB patients with respect to either healthy controls or AD with sensitivity from 87 to 100 % and specificity from 90 to 96 % depending on the stage of the disease.

**Conclusions:**

Our results are consistent with previous studies. We extend the scope of those studies by integrating prodromal DLB patients and by describing both hypo- and hyperperfusion in DLB. While decreases in perfusion may relate to functional impairments, increases might suggest a functional compensation of some brain areas.

**Electronic supplementary material:**

The online version of this article (doi:10.1186/s13195-016-0196-8) contains supplementary material, which is available to authorized users.

## Background

Dementia with Lewy bodies (DLB) is clinically characterized by cognitive impairment together with fluctuating cognition, parkinsonism, and visual hallucinations. It is the second most common form of neurodegenerative dementia after Alzheimer’s disease (AD), accounting for 10–20 % of patients with dementia [1].

A diagnosis of DLB is difficult to establish since at the early stage there is no clear cognitive pattern. Symptoms include non-amnestic mild cognitive impairment (MCI) [2] and at later stages DLB is mixed with other types of dementia. Establishing a differential diagnosis is all the more difficult as patients could present symptoms of AD, making DLB heterogeneous. Furthermore, clinical criteria lack sensitivity [1, 3, 4] and inter-rater reliability [5], although new neuropsychological tools have recently been developed [6, 7]. Therefore, in clinical routine, DLB can be mistaken for AD [3]. Yet, making an early and accurate diagnosis is mandatory for prognosis and management, as well as for pharmacological treatment [1], since some drugs such as neuroleptics have shown deleterious effects in DLB [8, 9].

Brain imaging may provide information to refine the diagnosis of DLB [10]. As functional changes can precede atrophy [11], functional neuroimaging may be particularly relevant to the study of the early stage of DLB. Among the various methods that assess brain functioning, cerebral blood flow (CBF) or brain perfusion can be evaluated using [15O] positron emission tomography (PET), single photon emission computerized tomography (SPECT), or arterial spin labeling (ASL) MRI. To our knowledge, brain perfusion in DLB has not been evaluated using [15O] PET. Studies using SPECT have shown occipital hypoperfusion [11–16] together with relatively preserved perfusion in the medial temporal lobe [14–16] in DLB in contrast to AD. The frontal, parietal, and cingulate cortices have also been reported to be altered when compared to elderly controls [15–17]. Similar results have been reported in ASL MRI studies [18–20] as well as in [18F]-fluoro-d-glucose PET studies assessing metabolism [21–27]. Taken together, these results indicate perfusion changes in DLB in the dementia stage. Therefore, we still have to assess early differences of perfusion between DLB and AD, at the prodromal stage (also named MCI).

Using magnetically labeled arterial blood water as an endogenous tracer, ASL has the advantage over SPECT and PET of being non-invasive, available in clinical routine, more reliable [28], and providing absolute values of CBF (i.e., without any global CBF weighting by the CBF of a given brain area, such as the cerebellum). ASL may, therefore, be a suitable tool for assessing perfusion in a diagnostic perspective.

In this study, we aimed to describe whole-brain ASL perfusion changes according to both the diagnosis and the cognitive impairment. We studied DLB and AD patients. For both diseases, patients were divided into a prodromal group and a mild dementia stage group, and we compared them with an elderly control group. We planned to assess differences between DLB and AD as well as between prodromal and the mild dementia stage in DLB. We postulated that ASL MRI would be sufficiently sensitive to detect changes in perfusion even in prodromal DLB. As global CBF (i.e., whole-brain CBF) seems to be altered in DLB [18], we also assessed regional CBF (rCBF, as relative perfusion) by a weighting of mean perfusion.

## Methods

### Participants

Altogether, 132 participants were recruited for this study, of which 12 patients were excluded due to motion during the MRI acquisition (see the section “MRI processing”), resulting in a total of 120 participants, comprising 44 patients with prodromal DLB (pro DLB group), 16 patients with DLB at the mild dementia stage (mild DLB group), 13 patients with prodromal AD (pro AD group), 26 patients with AD at the mild dementia stage (mild AD group), and 21 healthy elderly controls (HC group). Patients sharing both DLB and AD criteria were not included in the study. Demographic and clinical data are presented in Table 1. The five groups were examined by clinicians with expertise in dementia, who performed a complete anamnesis and medical examination. Using [29], akinesia, rigidity, and tremor at rest were rated from 0 to 4 (0 for no symptoms to 4 for serious impairment). Fluctuations were assessed with the Mayo Clinic Fluctuations scale [30] and the Newcastle-upon-Tyne Clinician Assessment of Fluctuation scale [31], and patients with a score greater than or equal to 2 were considered as having fluctuations. Cognitive functions were evaluated using the following tests:

- Mini-Mental State Examination (MMSE) for general cognitive functions

**Table 1:** Demographic and clinical data of participants

Characteristic	Pro DLB	Mild DLB	Pro AD	Mild AD	HC
Participants	46	16	13	25	21
Female	26	8	4 ^a^	17	12
Age, years (SD)	69.4 (8.8)	74.7 (10.2) ^∗^	74.5 (9.9) ^∗^	73.6 (9.1) ^∗^	64.8 (8.6)
MMSE score (SD) (maximum 30)	27.5 (1.4)	20.7 (3.4)	27.1 (1.5)	19.5 (3.4)	28.9 (1.0)
Participants with visual hallucinations	19	9	0	3	0
Participants with parkinsonism	34	11	1	7	0
Participants with cognitive fluctuations	28	9	0	5	0
Medial temporal lobe atrophy (L, R)					
Participants with a score of 0	18, 16	6, 4	3, 3	7, 5	13, 9
Participants with a score of 1	11, 12	1, 4	5, 6	5, 9	7, 10
Participants with a score of 2	12, 15	4, 4	4, 4	7, 5	1, 2
Participants with a score of 3	5, 2	3, 2	1, 0	5, 3	0, 0
Participants with a score of 4	0, 1	2, 2	0, 0	1, 3	0, 0
Parietal lobe atrophy (L, R)					
Participants with a score of 0	14, 14	9, 8	4, 2	5, 6	7, 8
Participants with a score of 1	16, 17	4, 5	7, 8	8, 6	9, 8
Participants with a score of 2	12, 11	3, 3	2, 3	9, 9	4, 5
Participants with a score of 3	0, 4	4, 0	0, 0	3, 4	0, 0
Participants with a score of 4	0, 0	0, 0	0, 0	0, 0	0, 0
Vascular damage in white matter					
Participants with a score of 0	25	4	7	9	7
Participants with a score of 1	15	7	4	7	12
Participants with a score of 2	6	4	1	7	2
Participants with a score of 3	0	1	1	2	0
Vascular damage in basal ganglia					
Participants with a score of 0	33	13	10	17	21
Participants with a score of 1	11	1	2	5	0
Participants with a score of 2	2	1	1	0	0
Participants with a score of 3	0	1	0	3	0
Participants with AchI medication	14	11	2	17	0
Participants with dopaminergic medication	13	6	0	0	0

Medial temporal lobe atrophy, parietal lobe atrophy, and vascular damage were assessed according to the Scheltens, Koedam, and Wahlund scales, respectively. Pro AD were more male than mild AD. Mild DLB, pro AD, and mild AD were older than HC *AchI* acetylcholinesterase inhibitor, *AD* Alzheimer’s disease, *DLB* dementia with Lewy bodies, *HC* healthy (elderly) controls, *L* left hemisphere, *MMSE* Mini-Mental State Examination, *R* right hemisphere, *SD* standard deviation

^*^p < 0.05

^a^p < 0.01
- The French version of the Free and Cued Selective Reminding Test, the Delayed Matching-to-Sample test (48 items), and the digit-span test for memory functions
- The *Dénomination Orale* (oral naming test, 80 items) for language
- The Frontal Assessment Battery, the Trail Making Test A and B, the Digit Symbol Substitution Test, and formal and semantic lexical evocation for executive functions
- The praxis set of Mahieux and the Rey-Osterrieth complex figure test for praxis
- The number localization and cube analysis of the Visual Object and Space Perception battery for visuo-perceptive functions

All patients underwent cerebrospinal fluid analysis, including measurement of tau, phospho-tau, and amyloid-beta (1–42) (Innogenetics’s Innotest *Ⓡ*, ELISA). Assessment of medial temporal lobe atrophy and parietal lobe atrophy on brain MRI were performed using the standardized Scheltens scale [32] and the Koedam scale [33] (5 categories, from 0 to 4 with 0 corresponding to no atrophy), respectively. Vascular damage in white matter and basal ganglia was assessed separately according to the Wahlund scale [34]. An etiologic diagnosis of the neurocognitive disorder for each patient was made using Dubois’s criteria for pro AD and mild AD [35], and McKeith’s criteria (probable DLB, i.e., at least two core symptoms) for mild DLB [1]. Pro DLB patients were defined as patients with MCI (Petersen criteria) [36], preservation of independence (assessed by the Instrumental Activities of Daily Living) and by McKeith’s criteria (meeting probable DLB criteria except presence of dementia) [1]. The numbers of patients treated with dopaminergic drugs or cholinesterase inhibitors are listed in Table 1. The control group consisted of elderly healthy and cognitively intact (no MCI) subjects who were recruited via advertisements in local community newsletters in Strasbourg, and via the listing of controls of the local clinical investigation center (Centre d’Investigation Clinique) in charge of any type of medical research of the University Hospital of Strasbourg. Exclusion criteria for participation in the study included contraindications for MRI, history of alcohol or substance misuse, evidence suggesting alternative neurological or psychiatric explanations for their symptoms or cognitive impairment, focal brain lesions on brain imaging, and the presence of other severe or unstable medical illness. All patients had a formal assessment of their diagnosis by three independent expert clinicians (FB, BC, and NP) and controls underwent similar clinical and cognitive assessments to exclude any who may have had occult MCI or dementia. Patients with concomitant AD and DLB, i.e., meeting both McKeith’s (for probable DLB) and Dubois’s criteria were also excluded.

The study was approved by the local Ethics Committee (Comité de Protections des Personnes Est IV, Strasbourg, France). Controls and patients gave written informed consent.

### Data acquisition

The pulsed ASL sequence was performed on a Siemens Verio 3T scanner equipped with a 32-channel head coil (Siemens, Erlangen, Germany). In total, 121 whole-brain T2 ^∗^-weighted (gradient echo) echo planar images were acquired using the QUIPPS II sequence provided by the manufacturer. The parameters were:

- Repetition time (TR): 3 s
- Flip angle: 90°
- Echo time (TE): 21 ms
- Inversion time 1 (TI1): 600 ms
- Inversion time 2 (TI2): 1325.1 ms
- Field of view (FOV): 152×256×112 mm
- Imaging matrix: 38×64×28
- 4 mm^3^ isotropic voxels, acceleration factor (generalized auto-calibrating partially parallel acquisitions [GRAPPA]): 2

The tagged volume was 10 cm thick, positioned at the neck, and its distal part was 23 mm below the first slice to avoid saturation. Bipolar gradients were used to eliminate the signals from fast moving spins (>10 cm ·*s*^−1^). The first volume recorded corresponded to M0, and non-tagged images were acquired in alternation with tagged images.

A 3D MPRAGE T1-weighted image was also acquired at the same session. The parameters were: imaging matrix 192×192×176 and 1 mm^3^ isotropic voxels.

### MRI processing

Images were processed using SPM8 (Welcome Department of Cognitive Neurology, London, UK) and in-house-developed software. All data were processed for each participant separately.

Functional images were first corrected for motion and magnetic field B0 inhomogeneities. According to the motion parameters provided by SPM, patients with translations and rotations higher than 2 mm and 2°, respectively, were removed from the analysis. As the TE is high enough to make the ASL sequence sensitive to blood oxygen level-dependent fluctuations, the signals were high-pass filtered at 0.1125 Hz according to the method of Chuang et al. [37]. One CBF map per subject was then calculated according to the TE-corrected method published by Foucher et al. [38]. The M0 map was coregistered to the T1 image and the transformation parameters were used to coregister the CBF map in the same way. The T1 image was segmented using the New Segment toolbox, leading to five high definition maps (gray and white matter, cerebrospinal fluid, meninges, and bones). The first three served as a brain mask to exclude non-cerebral voxels from the CBF map. Gray and white matter probability maps also served to calculate spatial normalization parameters to the Montreal National Institute (MNI) space according to the DARTEL approach. T1 and CBF maps were then spatially normalized according to the normalization parameters. During this procedure, CBF maps were smoothed (full width at half maximum of 8×8×8 mm) but not modulated.

### Statistical analyses

Analyses were performed in Matlab (R2012b, Mathworks, Natick, MA). The gender and age distribution between groups were assessed by chi-squared tests and a one-way ANOVA, respectively. A global decrease in perfusion in patients relative to healthy controls was evaluated by two-sample *T*-tests on values of whole-brain mean perfusion.

Voxel-wise statistical analyses of perfusion were performed using ANOVA, with age [39, 40] and gender [40] as regressors of non-interest. These analyses were both conducted in absolute and relative measurements, the latter being corrected for the global mean value. The relative measurement corrects for inter-individual differences in global perfusion and explains local differences in perfusion. The statistical analyses combined a *p*_uncorrected_<0.001 threshold at the voxel level with a cluster size threshold of 40 voxels (i.e., 320 mm^3^). Following a multiple comparison correction (familywise error or FWE) of *p*_FWE_<0.05 at the cluster level, significant clusters in the tables are identified by a superscript.

The clusters resulting from the voxel-wise analyses were subsequently considered as regions of interest (ROIs) in a two-class *k*-means classification and a linear discriminant analysis. The *k*-means analysis classified the patients and HC and provided the values of sensitivity and specificity for the patient group (or for DLB when the comparison concerned DLB versus AD). The discriminant analysis modeled the difference in perfusion between groups. As we aimed to model it with the simplest equation, we chose the shortest combination of ROIs that maximized the values of sensitivity and specificity (to assess for sensitivity and specificity, a leave-one-out cross-validation was used and the estimated classification was compared to the true diagnosis).

## Results

Demographic data are presented in Table 1. Age differed between groups (*F*[4,116])=4.4; *p*<0.01): mild DLB, pro AD, and mild AD being older than HC (*p*<0.05). The proportion of male patients was higher in the pro AD group than in the mild AD group (*p*<0.01).

### Perfusion in prodromal patients

The difference in global perfusion relative to HC was significant in the pro AD group (*p*<0.05) but not in the pro DLB group.

**Fig. 1:**
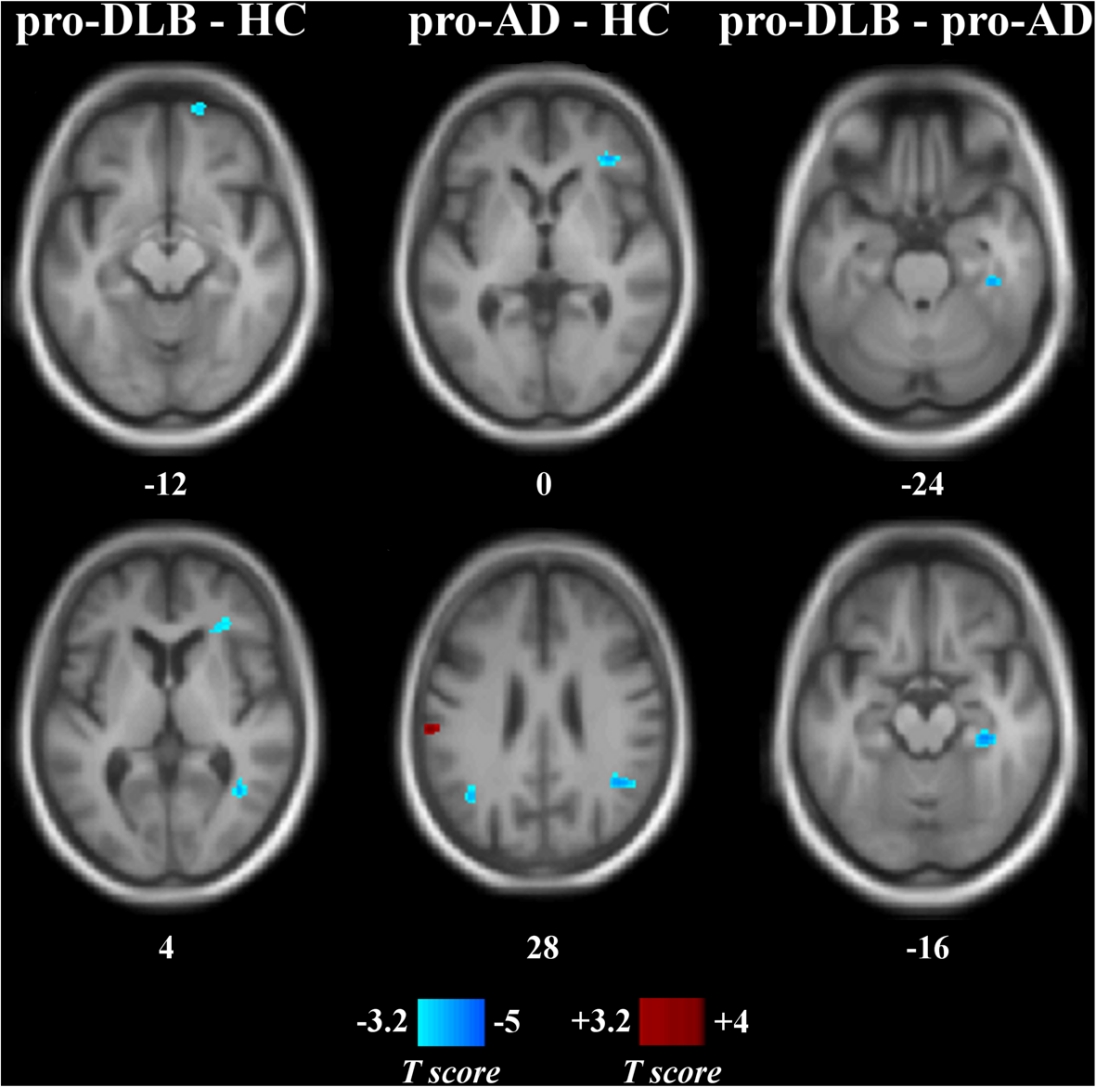
Statistical maps of relative perfusion in prodromal patients and healthy controls. Numbers are *z*-coordinates in the MNI space. *Left column*: Pro DLB minus HC. *Middle column*: Pro AD minus HC. *Right column*: Pro DLB minus pro AD. Positive (*red*) and negative (*blue*) *T*-values are, respectively, hyper- and hypoperfusion resulting from a voxel-wise ANOVA (*p*_uncorrected_<0.001, cluster size threshold of 40 voxels). The anatomical image used as a template is an average T1 from the encompassed groups. *AD* Alzheimer’s disease, *DLB* dementia with Lewy bodies, *HC* healthy (elderly) controls, *MNI* Montreal National Institute

The results for focal relative perfusion in prodromal patients are presented in Table 2 and Fig 1. Hypoperfusion in pro DLB compared to HC occurred in the right frontal, parietal, and temporal cortex together with the anterior insula. Only the left superior frontal gyrus showed an increase in relative perfusion in pro DLB. Absolute hypoperfusion gave similar results with a loss of perfusion in the right temporal and anterior insula, whereas the left superior frontal gyrus was hyperperfused (Additional file 1: Table S1 and Figure S1. Please refer to these supplementary data for all results of absolute perfusion). Pro AD showed hypoperfusion compared to HC in the right inferior frontal gyrus and bilateral angular gyrus, and hyperperfusion was seen in the left supramarginal gyrus (absolute assessment showed a similar pattern of hypoperfusion, but without any increase in perfusion relative to HC). Comparison between pro DLB and pro AD revealed a lower relative perfusion in DLB in the fusiform gyrus (as with absolute measurements). Pro AD did not have any hypoperfused brain areas relative to pro DLB (but absolute measurements showed a decrease in the left angular gyrus).

**Table 2:** Significant changes in relative perfusion in prodromal patients

Contrast	Region	Laterality	Extent	Coordinates (*x*,*y*,*z*)			Sensitivity	Specificity
Pro DLB < HC	Middle temporal	R	960	38	–58	6	65	76
	Anterior insula	R	840	36	34	0	74	90
	Inferior frontal							
	Superior parietal	R	648	16	–62	50	80	62
	Superior orbital	R	336	18	66	–14	54	95
Pro DLB > HC	Superior frontal	L	432	–16	32	38	57	90
Pro AD < HC	Angular	R	1280	42	–54	28	100	95
	Angular	L	448	–38	60	26	92	76
	Inferior frontal	R	400	34	38	0	76	92
Pro AD > HC	Supramarginal	L	432	–62	–24	26	69	90
Pro DLB < pro AD	Fusiform	R	984	34	–30	–18	72	69
Pro AD < pro DLB	–	–	–	–	–	–	–	–

Extent is expressed in mm^3^. Coordinates are in the Montreal National Institute space. Sensitivity and specificity are percentages, relative to DLB identification except for comparison between pro AD and HC *AD* Alzheimer’s disease, *DLB* dementia with Lewy bodies, *HC* healthy (elderly) controls, *L* left hemisphere, *R* right hemisphere

### Perfusion in patients with dementia

The difference in global perfusion relative to HC showed a trend towards statistical significance in mild AD (*p*=0.059), but not in mild DLB.

**Fig. 2:**
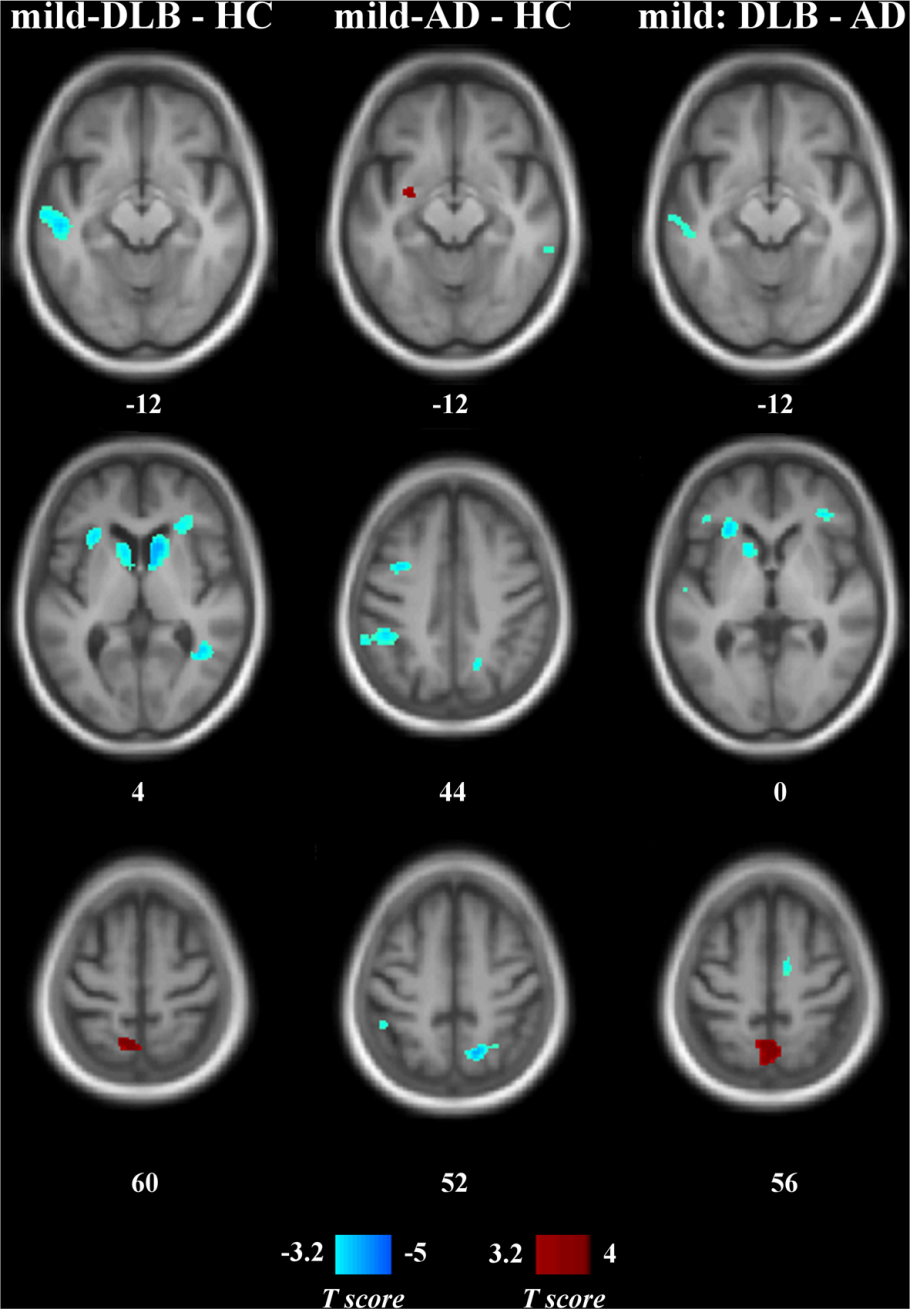
Statistical maps of relative perfusion in patients with mild dementia and healthy controls. Numbers are *z*-coordinates in the MNI space. *Left column*: Mild DLB minus HC. *Middle column*: Mild AD minus HC. *Right column*: Mild DLB minus mild AD. Positive (*red*) and negative (*blue*) *T*-values are, respectively, hyper- and hypoperfusion resulting from a voxel-wise ANOVA (*p*_uncorrected_<0.001, cluster size threshold of 40 voxels). The anatomical image used as a template is an average T1 from the encompassed groups. *AD* Alzheimer’s disease, *DLB* dementia with Lewy bodies, *HC* healthy (elderly) controls, *MNI* Montreal National Institute

Mild DLB patients showed a variety of hypoperfused brain areas compared to HC (Table 3 and Fig 2), including the frontal and temporal cortex, bilateral anterior insula, and caudate nucleus (as in absolute measurement; see Additional file 1: Table S2 and Figure S2). Hyperperfusion in mild DLB patients was observed mainly in the left precuneus. In mild AD, compared to HC, perfusion was reduced in parietal and temporal areas (assessment of absolute perfusion provided a similar pattern), whereas hyperperfusion was restricted to the left putamen (no increases in mild AD were revealed with absolute perfusion). When patient groups were compared, mild DLB showed a lower perfusion than mild AD in the frontal and temporal cortices together with the left supramarginal gyrus, anterior insula, and caudate nucleus (a difference in absolute perfusion only concerned the anterior insula and the supramarginal and superior temporal gyri). In contrast, mild AD had a reduced rCBF in the bilateral precuneus, left supramarginal, and medial superior frontal gyri (only the bilateral precuneus and the left supramarginal gyrus in assessment of absolute perfusion).

**Table 3:** Significant changes in relative perfusion in patients with mild dementia

Contrast	Region	Laterality	Extent	Coordinates (*x*,*y*,*z*)			Sensitivity	Specificity
Mild DLB < HC	Caudate nucleus ^∗^	R	5128	12	16	6	94	81
		L		–10	14	4		
	Middle temporal	L	2456	–54	–20	–14	81	86
	Middle temporal	R	2064	42	–52	8	75	76
	Anterior insula	R	1560	36	36	0	81	100
	Inferior frontal							
	Anterior insula	L	1072	–30	22	6	75	90
	Inferior frontal	L	576	–34	12	24	75	67
Mild DLB > HC	Precuneus	L	952	–6	–60	58	50	100
Mild AD < HC	Inferior parietal	L	1640	–46	–42	48	72	86
	Superior parietal	R	1440	16	–62	50	84	67
	Precuneus							
	Superior temporal	R	584	44	–48	18	84	76
	Precentral sulcus	L	472	–34	4	44	96	52
	Middle temporal	R	360	62	–42	14	72	81
	Superior temporal	L	352	–42	–42	0	80	76
Mild AD > HC	Putamen	L	352	–30	–4	–12	60	86
	Anterior insula	L	2168	–28	26	2	88	84
	Inferior frontal							
	Superior temporal	R	1416	–56	–18	4	94	88
	Caudate nucleus	L	1016	–14	16	–6	81	68
Mild DLB < mild AD	Inferior frontal	R	752	36	38	–2	88	76
	Supramarginal	L	752	–44	–28	34	88	72
	SMA	R	704	12	–6	54	69	92
	Inferior frontal	L	496	–38	12	22	63	80
	Middle temporal	L	480	–64	–20	–10	69	92
Mild AD < mild DLB	Precuneus ^∗^	LR	2896	2	–60	52	69	92
	Medial superior frontal	L	704	–2	60	32	69	92
	Medial superior frontal	L	584	–2	46	44	69	92
	Supramarginal	L	496	–56	–30	24	69	92

Extent is expressed in mm^3^. Coordinates are in the Montreal National Institute space. Sensitivity and specificity are percentages, relative to DLB identification except for comparison between mild AD and HC *AD* Alzheimer’s disease, *DLB* dementia with Lewy bodies, *HC* healthy (elderly) controls, *L* left hemisphere, *R* right hemisphere, *SMA* supplementary motor area

^*^ *p*_FWE_<0.05

### Perfusion according to the level of cognitive impairment

**Fig. 3:**
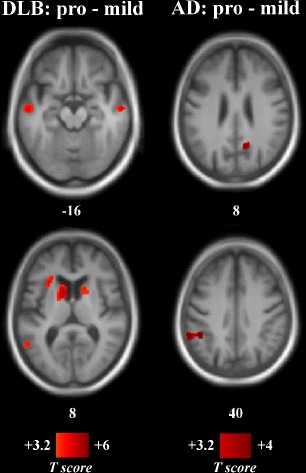
Statistical maps of relative perfusion according to the level of cognitive impairment. Numbers are *z*-coordinates in the MNI space. *Left column*: Pro DLB minus mild DLB. *Right column*: Pro AD minus mild AD. Positive (red) T-values are hypoperfusion in the mild groups resulting from a voxel-wise ANOVA (*p*_uncorrected_<0.001, cluster size threshold of 40 voxels). The anatomical image used as a template is an average T1 from the encompassed groups. *AD* Alzheimer’s disease, *DLB* dementia with Lewy bodies, *MNI* Montreal National Institute

Patients at different stages of the same disease were also compared, i.e., prodromal groups were compared to mild dementia groups. Mild DLB had lower perfusion than pro DLB in the left anterior insula, the inferior frontal gyrus, the right anterior and middle cingulum, the bilateral middle temporal gyrus, and the caudate nuclei (Table 4 and Fig. 3) (absolute perfusion revealed a similar pattern of decreasing perfusion; see Additional file 1: Table S3 and Figure S3). Mild AD had reduced perfusion compared to pro AD in the right precuneus and the left inferior parietal lobule (only the inferior parietal lobule was significant in measurement of absolute perfusion).

**Table 4:** Significant changes of relative perfusion according to the level of cognitive impairment

Contrast	Region	Laterality	Extent	Coordinates (*x*,*y*,*z*)		
Mild DLB < pro DLB	Caudate nucleus ^∗^	L	4968	–12	6	12
	Inferior frontal	L	2064	–32	14	24
	Middle temporal	L	1320	–58	–10	–18
	Anterior insula	L	1144	–28	24	4
	Caudate nucleus	R	936	10	10	4
	Middle cingulum	R	880	10	4	42
	Anterior cingulum	R	704	16	34	20
	Middle temporal	R	456	60	–12	–16
	Middle temporal	L	384	–56	–56	8
Pro DLB < mild DLB	Precuneus	L	744	–4	–60	58
Mild AD < pro AD	Inferior parietal	L	1544	–60	–46	42
	Precuneus	R	344	14	–60	28
Pro AD < mild AD	Cuneus	L	496	16	–102	12
	Inferior frontal	R	432	34	38	0

Extent is expressed in mm^3^. Coordinates are in the Montreal National Institute space. Sensitivity and specificity are percentages, relative to DLB identification except for comparison between AD and HC *AD* Alzheimer’s disease, *DLB* dementia with Lewy bodies, *HC* healthy (elderly) controls, *L* left hemisphere, *R* right hemisphere

^*^ *p*_FWE_<0.05

### Discriminant analysis

The linear discriminant analysis revealed the optimal weighting for the combination of ROIs that best segregated the two populations. We performed this analysis for all possible combinations of ROIs resulting from the voxel-wise comparisons. Since only cluster was significant between pro DLB and pro AD, no discriminant analysis was performed on this contrast. We obtained the most accurate classification compared to the medical diagnosis with the linear discriminant analysis formula shown in Table 5 (for discriminant analysis with absolute measurements of perfusion, see Additional file 1: Table S4). Values of sensitivity and specificity were good to perfect (Table 5). Nevertheless, they were higher when discriminating mild DLB than when discriminating pro DLB. No discriminant analysis was performed on the contrast between pro AD and HC since there was only one cluster.

**Table 5:** Linear discriminant analysis to classify subjects according to their relative pattern of perfusion

Contrast	Regions of interest	Coefficient	Constant	Sensitivity	Specificity
Pro DLB vs HC	Middle temporal (R)	5.5	–24.1	87	90
	Anterior insula/inferior frontal (R)	9.2			
	Superior parietal (R)	8.1			
	Superior orbitofrontal (R)	5.1			
	Superior frontal (L)	–5.9			
Mild DLB vs HC	Caudate nucleus (LR)	19.8	–38.5	94	95
	Middle temporal (R)	13.3			
	Anterior insula/inferior frontal (R)	4.8			
	Anterior insula (L)	8.4			
Mild DLB vs mild AD	Superior temporal (R)	9.2	–12.5	100	96
	Caudate nucleus (L)	5.1			
	Inferior frontal (L)	9.4			
	Precuneus (LR)	–2.8			
	Supramarginal (L)	–12.8			

Coefficients are mean coefficients (leave-one-out cross-validation) corrected for age and gender. As an example, to distinguish mild DLB from HC: Classification = constant + mean perfusion in bilateral caudate nucleus × 19.8 + mean perfusion in right middle temporal gyrus × 13.3 + mean perfusion in right anterior insula × 4.8 + mean perfusion in left anterior insula × 8.4 *AD* Alzheimer’s disease, *DLB* dementia with Lewy bodies, *HC* healthy (elderly) controls, *L* left hemisphere, *R* right hemisphere

## Discussion

Our aim was to describe brain perfusion disorders in the early stages of DLB using the pulsed ASL technique. We performed whole-brain voxel-wise analyses to compare two stages of DLB and AD, i.e., prodromal (or MCI) and dementia, as well as a fifth group of elderly controls. To evaluate whether perfusion MRI would help with diagnosis, we used classification tools (*k*-means and discriminant analysis) to assess the sensitivity and specificity of each significant cluster resulting from the voxel-wise analysis, and of specific combinations of clusters.

### Changes in DLB

Care is needed when interpreting differences in perfusion, particularly when two diseases or two stages of the same disease are being compared. Both hypo- and hyperperfusion were indeed observed in DLB relative to controls, suggesting distinct phenomena. Hypoperfusion occurred mainly in the middle temporal gyrus, and the right frontal and anterior insula in pro DLB; it then extended to the contralateral hemisphere and to the caudate nuclei when the disease worsened. This expansion of hypoperfused areas when the cognitive impairment increases was confirmed by contrasting pro DLB and mild DLB since it provided a similar pattern (plus the middle and anterior cingulum). Frontal and temporal hypoperfusion is consistent with previous reports [18, 20]. Hypoperfusion in prefrontal areas may be associated with impairment in DLB of executive functions [41] and attention [42], whereas the deficit in middle temporal areas would instead relate to memory [43]. A previous study on hallucination in AD [44] reported that the right anterior insula may be involved in the process of hallucination, suggesting that the early impairment of the anterior insula we described may be involved in the occurrence of hallucinations. The decrease in CBF in the caudate nuclei may instead be related to parkinsonism. According to previous studies, occipital hypoperfusion seems to be a core feature of DLB and may be related to visual hallucinations [14, 45]. Moreover, patients with DLB commonly present visual perceptual and visuospatial dysfunctions [46]. In our study, pro DLB and mild DLB did not show occipital hypoperfusion. However, a very strong effect of age was observed in the occipital cortex, which may mask the effect of the pathology and explain some discrepancies with previous studies, as most of them did not correct for age. The early stages of the disease and the number of patients who complain of visual hallucinations and visual impairment may also explain this difference [14]. Further correlates between perfusion and cognitive performances would provide information about variants in the first stage of DLB. In addition to the hypoperfusion pattern, the frontal cortex in pro DLB and the precuneus in mild DLB showed an increase in CBF compared to HC (contrasting pro DLB with mild DLB confirmed these results). Such observations are only partly due to the relative assessment of perfusion (by correcting for the whole-brain mean value), as absolute perfusion also revealed these hyperperfused regions. Whereas hypoperfusion relative to controls may reflect a decrease in neuronal activity in patients, hyperperfusion may reveal a compensatory process by which patients make up for the initial neuronal disorders [47–49]. Therefore, assessing perfusion seems to indicate a disorganization in DLB of some parts of the cortex, with both under- and over-activated areas.

### Changes in AD

Perfusion in AD was mainly reduced in the parietal and parietotemporal cortex, where the deficit started as early as the prodromal stage. Such impairments are in accordance with previous studies using ASL [50, 51], SPECT [13, 52], or PET [24]. Our data also showed that perfusion in these areas decreased with the cognitive decline (i.e., from the MCI stage to dementia). While our transversal study was not designed to compare the prodromal patients who convert to dementia to those who do not, the hypoperfusion we observed in the parietal and parietotemporal cortex and the fusiform gyrus has been reported by other groups as a predictor of conversion to dementia [53, 54]. Exploring perfusion according to the cognitive profile of patients with AD suggested that such hypoperfusion relates to the impairment of executive functions [55] and memory [56, 57]. We found that pro AD had lower perfusion than mild AD in the inferior frontal gyrus and the cuneus, which, according to our results and previous reports [15], is more typical of DLB. This counterintuitive result could reflect the lack of sensitivity of the DLB criteria [1, 3, 4], reinforcing the need for new biomarkers of DLB. Another explanation would be a compensatory phenomenon in mild DLB with hyperactivation in the frontal and occipital cortices.

### Changes between DLB and AD

Hypoperfusion in DLB relative to AD mainly occurred in the frontal cortex, the anterior insula, and the caudate nuclei, and in parts of the temporal cortex and supplementary motor area. Hypoperfusion in AD relative to DLB was in the medial frontal cortex and the parietal lobe. Hypoperfusion was unsurprisingly more extensive in dementia than in MCI, a finding that could be related to the stage of the disease but also to a lack of statistical power given the lower number of patients in the pro AD group. These patterns mainly agreed with the comparisons to HC. They are also in accordance with previous reports [18, 20] but differ in terms of occipital hypoperfusion in mild DLB (although Fong et al. [20] did not show occipital hypoperfusion either). The differences in perfusion between DLB and AD are consistent with the cognitive profiles of the diseases, the former being predominantly a frontal (and occipital) related disease and the latter being a posterior and medial temporal lobe related disease.

### Sensitivity and specificity

Discriminant analyses of the perfusion differences between DLB and HC and between DLB and AD provided good values of sensitivity and specificity, even with a small set of ROIs (sensitivity from 87 to 100 %; specificity from 90 to 96 %). These values were higher than those in previously published results obtained using SPECT [11, 12, 14, 15, 58] and PET [21, 24]. They were processed to give higher values than each ROI taken individually. Although mild DLB was more accurately classified than pro DLB, the good sensitivity and specificity suggest that DLB and AD could be differentiated at an early stage of the disease, such as the prodromal stage. It is noteworthy that the voxel-wise analysis and the discriminant analysis were performed on the same subjects since the sample size was too low to divide the groups into a learning and a testing data set. Therefore, the circular analysis we used can provide high values of sensitivity and specificity, which has to be considered as the upper limit of what can be achieved by a pulsed ASL study in discriminating DLB from HC and AD.

### Relative and absolute perfusion

Whole-brain CBF has been reported to be globally reduced in DLB [18]. Our results were not in line with this finding since AD but not DLB showed a global decrease of perfusion. This global reduction might mask some hyperactivation relative to whole-brain functioning and make the hypoperfusion pattern look more consistent that it actually is. By taking into account the mean perfusion, i.e., assessing relative perfusion as in SPECT, we described some local brain hypo- and hyperperfusion in DLB that was only partly observed in absolute values.

### Limitations

Some of the changes in perfusion observed in this study may partly be due to atrophy, even though we limited this bias by using the DARTEL normalization procedure (a common template across healthy subjects and patients was created) without modulation of the perfusion images (i.e., areas that were expanded or shrank to match the common template were not modified in intensity of perfusion). In this study, we assessed perfusion in DLB with a pulsed ASL sequence as did Taylor et al. [19], whereas other groups used pseudocontinuous [18] or continuous ASL [20]. Although the pseudocontinuous labeling technique has greater sensitivity than pulsed and continuous ASL, their intra- and mutli-center reproducibility is reasonable for the three ASL techniques [59]. Therefore, some differences between DLB, AD, and HC may still be hidden when using the pulsed ASL sequence, but the significant differences we found may not depend on the sequence. Another limitation of the study is the impact on statistical power due to differences in the number of participants between groups. As a consequence, some brain dysfunctions may have been underestimated. Other group differences could nevertheless be limited by the covariates: although the proportion of males was higher in the pro AD group than in the other groups, we accounted for gender in the statistical analyses, as we did for age. Lastly, although we used a leave-one-out cross-validation to classify the patients according to their perfusion pattern, a validation using other data sets will need to be performed. Nevertheless, this is one more step towards a multi-sequence and multi-MRI validation.

## Conclusions

This whole-brain voxel-wise study demonstrates that ASL can reveal specific changes in brain perfusion in DLB, even in its early stage such as in MCI. By combining perfusion values from a few ROIs, DLB can be differentiated from both AD and controls.

## Abbreviations

AchI, acetylcholinesterase inhibitor; AD, Alzheimer’s disease; ASL, arterial spin labeling; CBF, cerebral blood flow; DLB, dementia with Lewy bodies; FOV, field of view; FWE, familywise error; HC, healthy (elderly) controls; MCI, mild cognitive impairment; MMSE, Mini-Mental State Examination; MNI, Montreal National Institute; MRI, magnetic resonance imaging; PET, positron emission tomography; rCBF, regional cerebral blood flow; ROI, region of interest; SD, standard deviation; SMA, supplementary motor area; SPECT, single photo emission computerized tomography; TE, echo time; TI, inversion time; TR, repetition time

## Additional file

Additional file 1 Statistical maps, tables and linear discriminant analysis of absolute perfusion in patients and healthy controls. (PDF 2551 kb)

## References

[1] McKeith IG, Dickson DW, Lowe J, Emre M, O’Brien JT, Feldman H (2005). Diagnosis and management of dementia with Lewy bodies: third report of the DLB consortium. Neurology.

[2] Ferman TJ, Smith GE, Kantarci K, Boeve BF, Pankratz VS, Dickson DW (2013). Nonamnestic mild cognitive impairment progresses to dementia with Lewy bodies. Neurology.

[3] Nelson PT, Jicha Ga, Kryscio RJ, Abner EL, Schmitt Fa, Cooper G (2010). Low sensitivity in clinical diagnoses of dementia with Lewy bodies. J Neurol.

[4] Mega M, Masterman D, Benson D (1996). Dementia with Lewy bodies reliability and validity of clinical and pathologic criteria. Neurology.

[5] Richards M, Marder K (1991). Interrater reliability of extrapyramidal signs in a group assessed for dementia. Arch Neurol.

[6] Lee DR, McKeith I, Mosimann U, Ghosh-Nodial A, Grayson L, Wilson B (2014). The dementia cognitive fluctuation scale, a new psychometric test for clinicians to identify cognitive fluctuations in people with dementia. Am J Geriatr Psychiatr.

[7] Caffarra P, Gardini S, Dieci F, Copelli S, Maset L, Concari L (2013). The qualitative scoring MMSE pentagon test (QSPT): a new method for differentiating dementia with Lewy body from Alzheimer’s disease. Behav Neurol.

[8] Menendez-Gonzalez M, Calatayud MT, Blazquez-Menes B (2005). Exacerbation of Lewy bodies dementia due to memantine. J Alzheimer’s Dis.

[9] McKeith I, Fairbairn A, Perry R, Thompson P, Perry E (1992). Neuroleptic sensitivity in patients with senile dementia of Lewy body type. BMJ.

[10] Mak E, Su L, Williams G, O’Brien J (2014). Neuroimaging characteristics of dementia with Lewy bodies. Alzheimers Res Ther.

[11] Goto H, Ishii K, Uemura T, Miyamoto N, Yoshikawa T, Shimada K (2010). Differential diagnosis of dementia with Lewy bodies and Alzheimer disease using combined MR imaging and brain perfusion single-photon emission tomography. Am J Neuroradiol.

[12] Hanyu H, Shimizu S, Hirao K, Kanetaka H, Sakurai H, Iwamoto T (2006). Differentiation of dementia with Lewy bodies from Alzheimer’s disease using Mini-Mental State Examination and brain perfusion SPECT. J Neurol Sci.

[13] Colloby SJ, Fenwick JD, Williams ED, Paling SM, Lobotesis K, Ballard C (2002). A comparison of (99m)Tc-HMPAO SPET changes in dementia with Lewy bodies and Alzheimer’s disease using statistical parametric mapping. Eur J Nucl Med Mol Imaging.

[14] Pasquier J, Michel BF, Brenot-Rossi I, Hassan-Sebbag N, Sauvan R, Gastaut JL (2002). Value of (99m)Tc-ECD SPET for the diagnosis of dementia with Lewy bodies. Eur J Nucl Med Mol Imaging.

[15] Lobotesis K, Fenwick J, Phipps A, Ryman A, Swann A, Ballard C (2001). Occipital hypoperfusion on SPECT in dementia with Lewy bodies but not AD. Neurology.

[16] Ishii K, Yamaji S, Kitagaki H, Imamura T, Hirono N, Mori E (1999). Regional cerebral blood flow difference between dementia with Lewy bodies and AD. Neurology.

[17] Takahashi R, Ishii K, Shimada K, Ohkawa S, Nishimura Y (2010). Hypoperfusion of the motor cortex associated with parkinsonism in dementia with Lewy bodies. J Neurol Sci.

[18] Binnewijzend Maa, Kuijer JPa, Benedictus MR, Möller CM, Pijnenburg YaL, van der Flier. WM (2014). Distinct perfusion patterns in Alzheimer’s disease, frontotemporal dementia and dementia with Lewy bodies. Eur Radiol.

[19] Taylor JP, Firbank MJ, He J, Barnett N, Pearce S, Livingstone A (2012). Visual cortex in dementia with Lewy bodies: magnetic resonance imaging study. Br J Psychiatry J Ment Sci.

[20] Fong T, Inouye S, Dai W, Press D, Alsop D (2011). Association cortex hypoperfusion in mild dementia with Lewy bodies: a potential indicator of cholinergic dysfunction?. Brain Imaging Behav.

[21] O’Brien JT, Firbank MJ, Davison C, Barnett N, Bamford C, Donaldson C (2014). 18F-FDG PET and perfusion SPECT in the diagnosis of Alzheimer and Lewy body dementias. J Nucl Med.

[22] Spehl TS, Hellwig S, Amtage F, Weiller C, Bormann T, Weber WA (2015). Syndrome-specific patterns of regional cerebral glucose metabolism in posterior cortical atrophy in comparison to dementia with Lewy bodies and Alzheimer’s disease – a [F-18]-FDG PET study. J Neuroimaging.

[23] Kantarci K, Lowe V, Boeve B, Weigand S, Senjem M, Przybelski S (2012). Multimodality imaging characteristics of dementia with Lewy bodies. Neurobiol Aging.

[24] Gilman S, Koeppe RA, Little R, An H, Junck L, Giordani B (2005). Differentiation of Alzheimer’s disease from dementia with Lewy bodies utilizing positron emission tomography with [18F]fluorodeoxyglucose and neuropsychological testing. Exp Neurol.

[25] Ishii K, Soma T, Kono AK, Sofue K, Miyamoto N, Yoshikawa T (2007). Comparison of regional brain volume and glucose metabolism between patients with mild dementia with Lewy bodies and those with mild Alzheimer’s disease. J Nucl Med.

[26] Ishii K, Imamura T, Sasaki M, Yamaji S, Hirono N, Shimomura T (1998). Regional cerebral glucose metabolism in dementia with Lewy bodies and Alzheimer’s disease. Neurology.

[27] Imamura T, Ishii K, Sasaki M, Kitagaki H, Yamaji S, Hirono N (1997). Regional cerebral glucose metabolism in dementia with Lewy bodies and Alzheimer’s disease: a comparative study using positron emission tomography. Neurosci Lett.

[28] Henriksen OM, Larsson HBW, Hansen AE, Grüner JM, Law I, Rostrup E (2012). Estimation of intersubject variability of cerebral blood flow measurements using MRI and positron emission tomography. J Magn Reson Imaging.

[29] The Unified Parkinson’s Disease Rating Scale (UPDRS): Status and recommendations. Mov. Disord. 2003; 18:738–750. doi:http://dx.doi.org/10.1002/mds.10473. 10.1002/mds.10473 12815652

[30] Ferman TJ, Smith GE, Boeve BF, Ivnik RJ, Petersen RC, Knopman D (2004). DLB fluctuation. Specific features that reliably differentiate dementia with Lewy bodies from Alzheimer’s disease and normal aging. Neurology.

[31] Walker MP, Ayre GA, Cummings JL, Wesnes K, McKeith IG, O’Brien JT (2000). The clinician assessment of fluctuation and the one day fluctuation assessment scale: two methods to assess fluctuating confusion in dementia. Br J Psychiatry.

[32] Scheltens P, Launer L, Barkhof F, Weinstein H, van Gool W (1995). Visual assessment of medial temporal lobe atrophy on magnetic resonance imaging: interobserver reliability. J Neurol.

[33] Koedam ELGE, Lehmann M, Van Der Flier WM, Scheltens P, Pijnenburg YAL, Fox N (2011). Visual assessment of posterior atrophy development of a MRI rating scale. Eur Radiol.

[34] Wahlund LO, Barkhof F, Fazekas F, Bronge L, Augustin M, Sjögren M (2001). A new rating scale for age-related white matter changes. Stroke.

[35] Dubois B, Feldman HH, Jacova C, Dekosky ST, Barberger-Gateau P, Cummings J (2007). Research criteria for the diagnosis of Alzheimer’s disease: revising the NINCDS-ADRDA criteria. Lancet Neurol.

[36] Petersen RC (2004). Mild cognitive impairment as a diagnostic utility. J Intern Med.

[37] Chuang K, Gelderen PV, Merkle H, Bodurka J, Ikonomidou V, Koretsky A (2008). Mapping resting-state functional connectivity using perfusion MRI. Neuroimage.

[38] Foucher JR, Roquet D, Marrer C, Pham BT, Gounot D (2011). Correcting for the echo-time effect after measuring the cerebral blood flow by arterial spin labeling. J Magn Reson Imaging.

[39] Preibisch C, Sorg C, Förschler A, Grimmer T, Sax I, Wohlschläger AM (2011). Age-related cerebral perfusion changes in the parietal and temporal lobes measured by pulsed arterial spin labeling. J Magn Reson Imaging.

[40] Liu Y, Zhu X, Feinberg D, Guenther M, Gregori J, Weiner MW (2012). Arterial spin labeling MRI study of age and gender effects on brain perfusion hemodynamics. Magn Reson Medicine Off J Soc Magn Reson Med / Soc Magn Reson Med.

[41] Yoon JH, Kim M, Moon SY, Yong SW, Hong JM (2015). Olfactory function and neuropsychological profile to differentiate dementia with Lewy bodies from Alzheimer’s disease in patients with mild cognitive impairment: a 5-year follow-up study. J Neurol Sci.

[42] Ferman TJ, Smith GE, Boeve BF, Graff-Radford NR, Lucas JA, Knopman DS (2006). Neuropsychological differentiation of dementia with Lewy bodies from normal aging and Alzheimer’s disease. Clin Neuropsychol.

[43] Mondon K, Gochard A, Marqué A, Armand A, Beauchamp D, Prunier C (2007). Visual recognition memory differentiates dementia with Lewy bodies and Parkinson’s disease dementia. J Neurol Neurosurg Psychiatry.

[44] Blanc F, Noblet V, Philippi N, Cretin B, Foucher J, Armspach JP (2014). Right anterior insula: core region of hallucinations in cognitive neurodegenerative diseases. PloS ONE.

[45] Heitz C, Noblet V, Cretin B, Philippi N, Kremer L, Stackfleth M (2015). Neural correlates of visual hallucinations in dementia with Lewy bodies. Alzheimer’s Res Ther.

[46] Cagnin A, Gnoato F, Jelcic N, Favaretto S, Zarantonello G, Ermani M (2013). Clinical and cognitive correlates of visual hallucinations in dementia with Lewy bodies. J Neurol Neurosurg Psychiatry.

[47] Park D, Reuter-Lorenz P (2009). The adaptive brain: aging and neurocognitive scaffolding. Annu Rev Psychol.

[48] Persson J, Nyberg L (2006). Altered brain activity in healthy seniors: what does it mean?. Prog Brain Res.

[49] Cabeza R, Anderson ND, Locantore JK, McIntosh AR (2002). Aging gracefully: compensatory brain activity in high-performing older adults. NeuroImage.

[50] Binnewijzend MAA, Benedictus MR, Kuijer JPA, van der Flier WM, Teunissen CE, Prins ND (2016). Cerebral perfusion in the predementia stages of Alzheimer’s disease. Eur Radiol.

[51] Alexopoulos P, Sorg C, Förschler A, Grimmer T, Skokou M, Wohlschläger A (2012). Perfusion abnormalities in mild cognitive impairment and mild dementia in Alzheimer’s disease measured by pulsed arterial spin labeling MRI. Eur Arch Psychiatry Clin Neurosci.

[52] Habert MO, Horn JF, Sarazin M, Lotterie JA, Puel M, Onen F (2011). Brain perfusion SPECT with an automated quantitative tool can identify prodromal Alzheimer’s disease among patients with mild cognitive impairment. Neurobiol Aging.

[53] Chao LL, Buckley ST, Kornak J, Schuff N, Madison C, Yaffe K (2010). ASL perfusion MRI predicts cognitive decline and conversion from MCI to dementia. Alzheimer Dis Assoc Disord.

[54] Caroli A, Testa C, Geroldi C, Nobili F, Barnden LR, Guerra UP (2007). Cerebral perfusion correlates of conversion to Alzheimer’s disease in amnestic mild cognitive impairment. J Neurol.

[55] Chao LL, Pa J, Duarte A, Schuff N, Weiner MW, Kramer JH (2009). Patterns of cerebral hypoperfusion in amnestic and dysexecutive MCI. Alzheimer Dis Assoc Disord.

[56] Nobili F, Frisoni GB, Portet F, Verhey F, Rodriguez G, Caroli A (2008). Brain SPECT in subtypes of mild cognitive impairment: findings from the DESCRIPA multicenter study. J Neurol.

[57] Nobili F, Brugnolo A, Calvini P, Copello F, De Leo C, Girtler N (2005). Resting SPECT–neuropsychology correlation in very mild Alzheimer’s disease. Clin Neurophysiol.

[58] Colloby SJ, Taylor JP, Davison CM, Lloyd JJ, Firbank MJ, McKeith IG (2013). Multivariate spatial covariance analysis of SPECT images in dementia with Lewy bodies and AD: utility in differential diagnosis. J Cereb Blood Flow Metab.

[59] Gevers S, Van Osch MJ, Bokkers RP, Kies DA, Teeuwisse WM, Majoie CB (2011). Intra- and multicenter reproducibility of pulsed, continuous and pseudo-continuous arterial spin labeling methods for measuring cerebral perfusion. J Cereb Blood Flow Metab.

