# Supplementary material for: Brain perfusion in dementia with Lewy bodies and Alzheimer’s disease: an arterial spin labeling MRI study on prodromal and mild dementia stages
Source: Alzheimers Res Ther. 2016 Jul 12;8:29. doi: 10.1186/s13195-016-0196-8 (PMC4940880; doi:10.1186/s13195-016-0196-8)
Supplement: Additional file 1 — Statistical maps, tables and linear discriminant analysis of absolute perfusion in patients and healthy controls. (PDF 2551 kb) [file 13195_2016_196_MOESM1_ESM.pdf]

Table 1. Significant changes in absolute perfusion in prodromal patients

Contrast	Region	Laterality	Extent	Coordinates (x,y,z)			Sensitivity	Specificity
pro-DLB < HC	Middle temporal	R	760	38	-56	8	72	81
	Anterior insula Inferior frontal	R	432	36	34	0	62	90
pro-DLB > HC	Superior frontal	L	456	-16	32	38	92	62
pro-AD < HC	Angular	R	1712	40	-50	8	92	95
	Anterior insula Inferior frontal	R	536	36	36	0	100	71
	Angular	L	416	-38	-60	26	92	62
pro-AD > HC	-	-	-	-	-	-	-	-
pro-DLB < pro-AD	Fusiform	R	840	34	-30	-18	72	69
pro-AD < pro-DLB	Angular	L	424	-46	-54	30	76	85
	Angular	L	360	-38	-62	24	67	69

L and R refer to left and right hemisphere, respectively. Extent is expressed in mm^3. Coordinates are in the MNI space. Sensitivity and specificity are in percentage, relative to DLB identification except for comparison between pro-AD and HC.

Figure 1. Statistical maps of absolute perfusion in prodromal patients and healthy controls

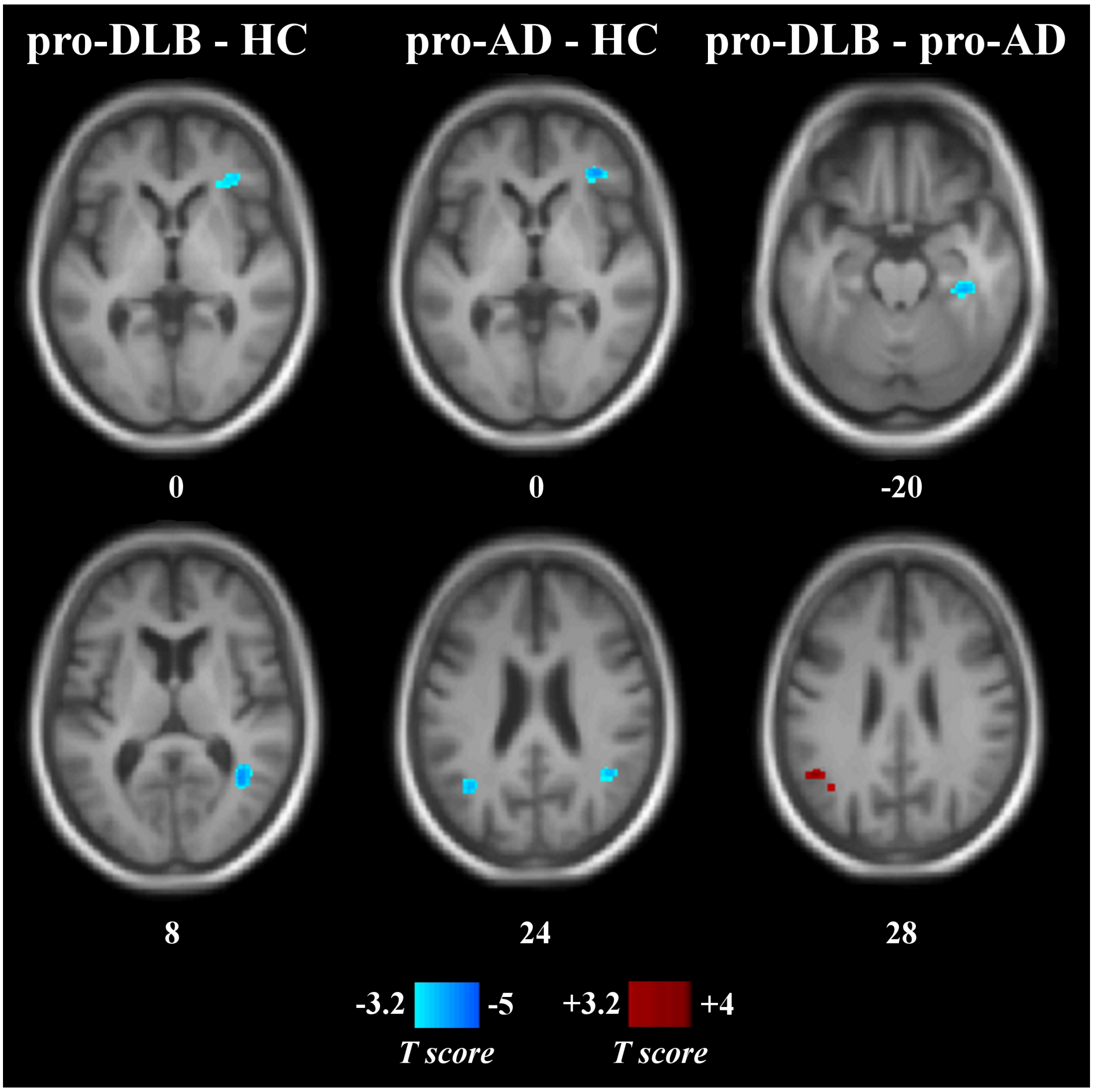

Numbers are z coordinates in the MNI-space. Left panel: pro-DLB minus HC; middle panel: pro-AD minus HC; right panel: pro-DLB minus pro-AD. Positive (red) T-values are respectively hyper- and hypoperfusion resulting from a voxel-wise ANOVA ($p_{uncorrected} < 0.001$, cluster size threshold of 320 mm3). Anatomical image used as template is an average T1 from the encompassed groups.

Table 2. Significant changes in absolute perfusion in patients with mild dementia.

Contrast	Region	Laterality	Extent	Coordinates (x,y,z)			Sensitivity	Specificity
mild-DLB < HC	Middle temporal	R	1896	42	-52	8	88	90
	Middle temporal	L	1880	-54	-20	-14	88	86
	Caudate	R	1488	10	16	6	81	57
	Anterior insula<br>Inferior frontal	R	1120	36	36	0	81	67
	Caudate	L	496	-10	12	4	81	76
	Anterior insula<br>Inferior frontal	L	480	-30	22	6	75	86
	Inferior frontal	L	376	-34	12	24	100	57
mild-DLB > HC	Precuneus	L	952	-6	-60	58	56	81
mild-AD < HC	Inferior parietal	L	1640	-46	-42	46	68	95
	Superior pairetal<br>Precuneus	R	1440	16	-62	50	84	86
	Middle temporal	R	360	62	-44	-16	72	81
	Superior temporal	R	584	44	-50	16	84	52
	Precentral	L	472	-34	4	44	88	52
	Superior temporal	L	344	-42	-42	0	72	71
mild-AD > HC	-	-	-	-	-	-	-	-
mild-DLB < mild-AD	Anterior insula<br>Inferior frontal	L	504	-26	28	2	75	72
	Supramarginal	L	496	-44	-28	34	84	64
	Superior temporal	L	440	-56	-18	4	81	88
mild-DLB < mild-AD	Precuneus	LR	2288	0	-66	58	75	76
	Supramarginal	L	536	-56	-30	24	63	80

L and R refer to left and right hemisphere, respectively. Extent is expressed in mm^3. Coordinates are in the MNI space. Sensitivity and specificity are in percentage, relative to DLB identification except for comparison between mild-AD and HC.

Figure 2. Statistical maps of absolute perfusion in patients with mild dementia and healthy controls.

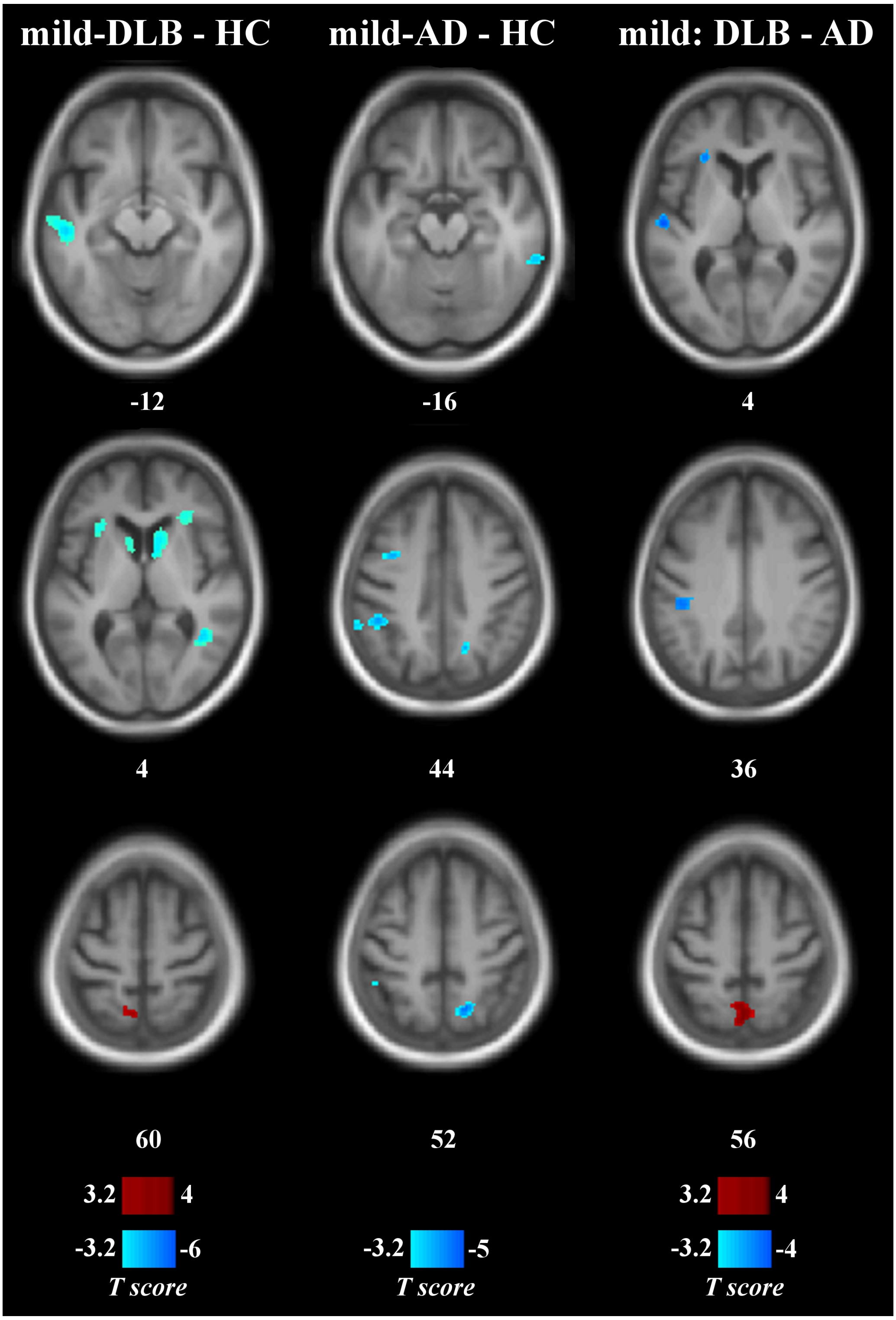

Numbers are z coordinates in the MNI-space. Left panel: mild-DLB minus HC; middle panel: mild-AD minus HC; right panel: mild-DLB minus mild-AD. Positive (red) T-values are hypoperfusion in the mild groups resulting from a voxel-wise ANOVA ($p_{uncorrected} < 0.001$, cluster size threshold of 320 mm3). Anatomical image used as template is an average T1 from the encompassed groups.

Table 3. Significant changes of absolute perfusion according to the level of cognitive impairment.

Contrast	Region	Laterality	Extent	Coordinates (x,y,z)		
pro-DLB < mild-DLD	-	-	-	-	-	
mild-DLD < pro-DLB	Caudate *	L	3792	-10	12	2
	Inferior frontal	L	2024	-34	14	24
	Middle temporal	L	1352	-56	-12	-18
	Postcentral sulcus	R	1136	34	-40	38
	Anterior insula	L	848	-28	26	4
	Middle cingulm	R	600	12	4	40
	Anterior cingulum	R	440	16	32	22
	Middle temporal	R	360	60	-12	-16
pro-AD < mild-AD	-	-	-	-	-	-
mild-AD < pro-AD	Inferior parietal	L	1136	-44	-44	36

*, $p_{FWE} < 0.05$. L and R refer to left and right hemisphere, respectively. Extent is expressed in mm^3. Coordinates are in the MNI space. Sensitivity and specificity are in percentage, relative to DLB identification except for comparison between AD and HC.

Figure 3. Statistical maps of absolute perfusion according to the level of cognitive impairment.

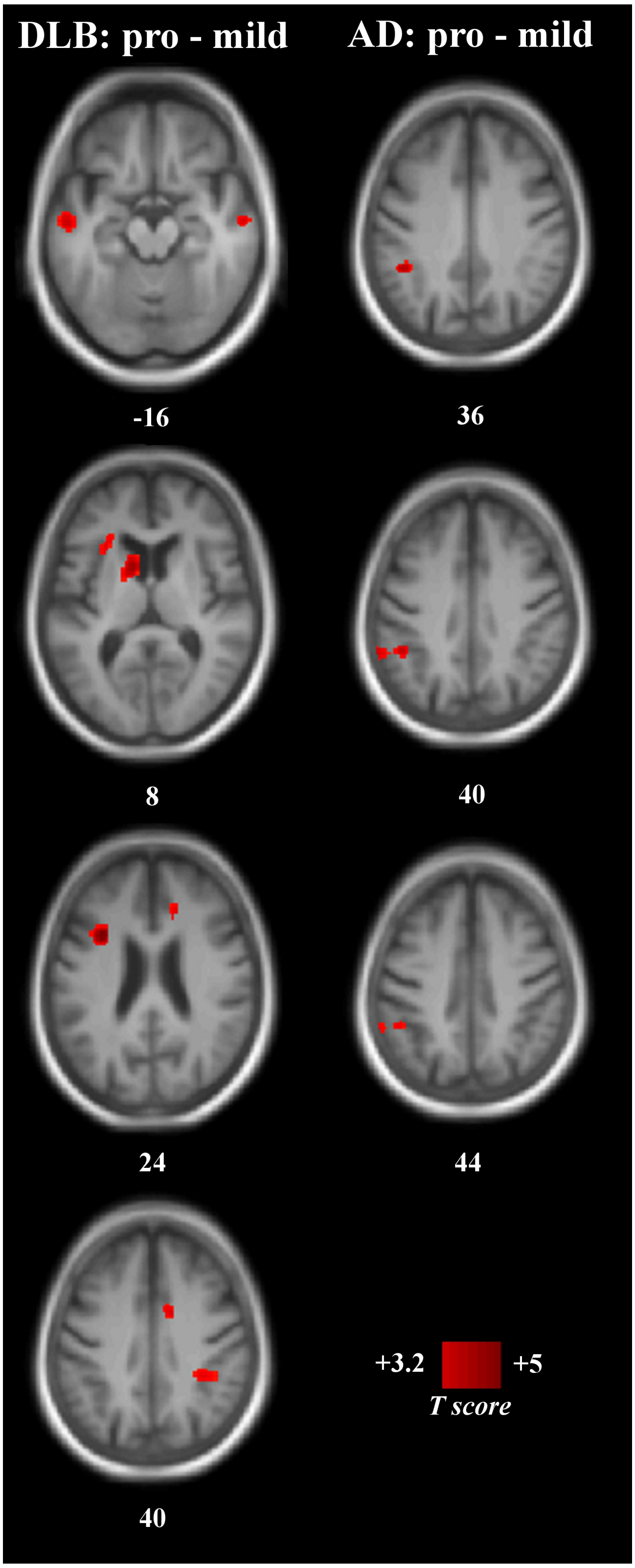

Numbers are z coordinates in the MNI-space. Left pannel: pro-DLB versus mild-DLB; right pannel: pro-AD versus mild-AD. Positive (red) T-values are hypoperfusion in the mild groups resulting from a voxel-wise ANOVA ($p_{uncorrected} < 0.001$, cluster size threshold of 320 mm3). Anatomical image used as template is an average T1 from the encompassed groups.

Table 4. Linear discriminant analysis to classify subjects according to their absolute pattern of perfusion.

Contrast	Region	Coefficient	Constant	Sensitivity	Specificity
pro-DLB vs HC	Middle temporal (R)<br>Anterior insula / Inferior frontal (R)<br>Superior frontal (L)	0.11<br>0.10<br>-0.11	-5.1	80	86
pro-AD vs HC	Angular gyrus (R)<br>Anterior insula / Inferior frontal (R)	0.22<br>0.11	-17.1	92	90
pro-DLB vs pro-AD	Fusiform (R)<br>Angular (L)	0.12<br>-0.08	-1.25	85	92
mild-DLB vs HC	Middle temporal (L)<br>Precuneus (L)	0.17<br>-0.12	-5.9	88	95
mild-AD vs HC	Inferior parietal (L)<br>Precentral (L)<br>Precuneus (R)	0.08<br>0.04<br>0.10	-16.4	84	86
mild-DLB vs mild-AD	Supramarginal (L)<br>Superior temporal (L)<br>Supramarginal (L)	0.16<br>0.13<br>-0.31	-2.51	100	88

L and R refer to left and right hemisphere, respectively. Coefficients are mean coefficients (leave-one-out cross-validation) corrected for age and gender. As an example, to distinguish mild-DLB from HC, Classification = constant + "mean perfusion in right middle temporal gyrus" × 0.17 + "mean perfusion in left precuneus" × -0.12.
